# 215a. Use of Viracor Cytomegalovirus T-Cell Immunity Panel (TCIP) to Predict the Risk of Recurrent Cytomegalovirus Infection in Solid Transplant Patients and Guide Appropriate Management

**DOI:** 10.1093/ofid/ofac492.292

**Published:** 2022-12-15

**Authors:** Indre Vysniauskaite, Kexin Guo, Michael P Angarone, Valentina Stosor, Sajal D Tanna, Michael G Ison

**Affiliations:** Northwestern University Feinberg School of Medicine, Chicago, Illinois; Northwestern University Feinberg School of Medicine, Chicago, Illinois; Northwestern University Feinberg School of Medicine, Chicago, Illinois; Northwestern University Feinberg School of Medicine, Chicago, Illinois; Northwestern University Feinberg School of Medicine, Chicago, Illinois; Northwestern University Feinberg School of Medicine, Chicago, Illinois

## Abstract

**Background:**

Cytomegalovirus infection (CMV) is the most common opportunistic infection in solid organ transplant recipients (SOTR). Predicting recurrent disease and appropriate use of secondary antiviral prophylaxis is currently poorly defined. CMV-specific T-cell assays have been shown to be promising tools in determining presence of CMV immunity and helping guide primary and secondary antiviral prophylaxis. The goal of this study was to evaluate the ability of commercially available CMV-TCIP in predicting the risk of CMV relapse.

**Methods:**

After IRB approval, clinical and laboratory data was retrospectively collected from our Enterprise Data Warehouse and primary chart review on 74 SOTR. As part of routine clinical care, the CMV T-Cell Immunity Panel (CMV-TCIP, Viracor Eurofins, Lenexa, KS) were collected by the TID provider managing the infection; generally this was done around the time of first negative test to inform choice of 2° prophylaxis. Analysis was completed for the total patient population and a subgroup of patients with CMV-TCIP tests completed ± 21 days of the first negative CMV VL. Descriptive statistics and Cohen’s Kappa statistics were used to assess association of relapse with assay results.

**Results:**

Patient demographics are shown in Table 1. 2° prophylaxis was given to 60 (81.1%) of total patients for a mean of 86 days (0-614 days) after the first negative CMV VL. 26 (35.1%) SOTRs had relapse – 9 with positive CD4 and 20 with positive CD8 CMV-TCIP values. There was no association between specific test results and risk of relapse in the overall study population (see table 2). Similarly, no significance was found in the subgroup analysis of patients with CMV-TCIP results ± 21 days after first negative CMV VL.

**Figure:**
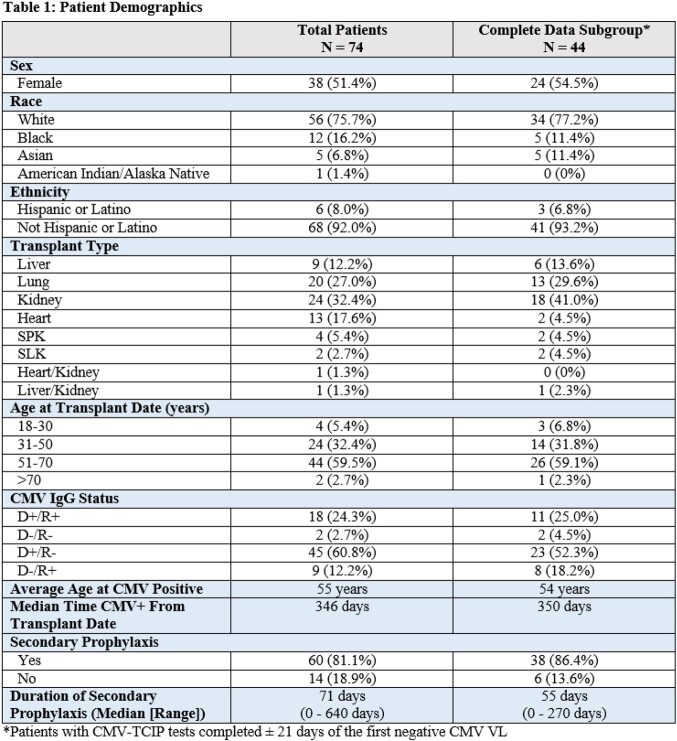

**Figure:**
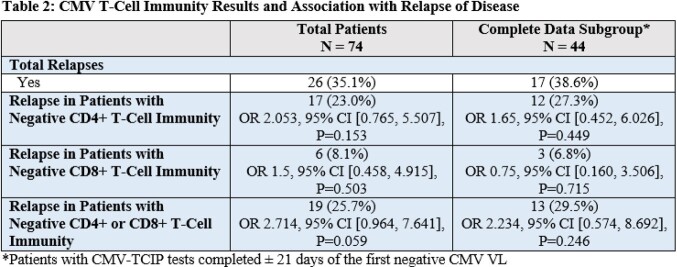

**Conclusion:**

In this study, CMV-TCIP did not predict CMV relapse after initial infection and treatment. While prior studies have shown a potential role for assessment of CMV-specific immunity to guide need for secondary prophylaxis, more studies of the CMV-TCIP are needed to assess the utility of this assay for this indication.

**Disclosures:**

**Michael P. Angarone, DO**, Abbvie: Advisor/Consultant **Valentina Stosor, MD**, DiaSorin: Advisor/Consultant|Eli Lilly and Company: Grant/Research Support|Med Learning Group: Honoraria **Michael G. Ison, MD MS**, GlaxoSmithKlein: Grant/Research Support|Pulmocide: Grant/Research Support|Viracor Eurfins: Advisor/Consultant.

